# Discovering Sequence Motifs with Arbitrary Insertions and Deletions

**DOI:** 10.1371/journal.pcbi.1000071

**Published:** 2008-05-09

**Authors:** Martin C. Frith, Neil F. W. Saunders, Bostjan Kobe, Timothy L. Bailey

**Affiliations:** 1 Computational Biology Research Center, National Institute of Advanced Industrial Science and Technology (AIST), Tokyo, Japan; 2 School of Molecular and Microbial Sciences, University of Queensland, Brisbane, Queensland, Australia; 3 Institute for Molecular Bioscience, University of Queensland, Brisbane, Queensland, Australia; Washington University, United States of America

## Abstract

Biology is encoded in molecular sequences: deciphering this encoding remains a grand scientific challenge. Functional regions of DNA, RNA, and protein sequences often exhibit characteristic but subtle motifs; thus, computational discovery of motifs in sequences is a fundamental and much-studied problem. However, most current algorithms do not allow for insertions or deletions (indels) within motifs, and the few that do have other limitations. We present a method, GLAM2 (Gapped Local Alignment of Motifs), for discovering motifs allowing indels in a fully general manner, and a companion method GLAM2SCAN for searching sequence databases using such motifs. glam2 is a generalization of the gapless Gibbs sampling algorithm. It re-discovers variable-width protein motifs from the PROSITE database significantly more accurately than the alternative methods PRATT and SAM-T2K. Furthermore, it usefully refines protein motifs from the ELM database: in some cases, the refined motifs make orders of magnitude fewer overpredictions than the original ELM regular expressions. GLAM2 performs respectably on the BAliBASE multiple alignment benchmark, and may be superior to leading multiple alignment methods for “motif-like” alignments with N- and C-terminal extensions. Finally, we demonstrate the use of GLAM2 to discover protein kinase substrate motifs and a gapped DNA motif for the LIM-only transcriptional regulatory complex: using GLAM2SCAN, we identify promising targets for the latter. GLAM2 is especially promising for short protein motifs, and it should improve our ability to identify the protein cleavage sites, interaction sites, post-translational modification attachment sites, etc., that underlie much of biology. It may be equally useful for arbitrarily gapped motifs in DNA and RNA, although fewer examples of such motifs are known at present. GLAM2 is public domain software, available for download at http://bioinformatics.org.au/glam2.

## Author Summary

In recent decades, scientists have extracted genetic sequences—DNA, RNA, and protein sequences—from numerous organisms. These sequences hold the information for the construction and functioning of these organisms, but as yet we are mostly unable to read them. It has long been known that these sequences contain many kinds of “motifs”, i.e. re-occurring patterns, associated with specific biological functions. Thus, much research has been devoted to computer algorithms for automatically discovering subtle, recurring motifs in sequences. However, previous algorithms search for rigid motifs whose instances vary only by substitutions, and not by insertions or deletions. Real motifs are flexible, and do vary by insertions and deletions. This study describes a new computer algorithm for discovering motifs, which allows for arbitrary insertions and deletions. This algorithm can discover real, flexible motifs, and should be able to help us determine the functions of many biological molecules.

## Introduction

Sequence motifs are important tools in molecular biology. Sequence motifs can describe and identify features in DNA, RNA and protein sequences such as transcription factor binding sites, splice junctions and protein-protein interaction sites. Numerous algorithms have been developed for discovering motifs, as well as algorithms for scanning databases for matches to a given motif or motifs. Some are specialized for discovery of DNA motifs. These include A-GLAM [1], AlignACE [2], BioProspector [3], MDscan [4], RSA Tools [5],[6], Weeder [7] and YMF [8]. Others, such as MEME [9] and Gibbs [10] can discover motifs in either protein or DNA sequences. The importance of motifs is further underscored by the numerous databases that have been compiled of known motifs including DNA regulatory motifs in TRANSFAC, JASPAR, SCPD, DBTBS, RegulonDB [11]–[14], and protein motifs in ELM, PROSITE, BLOCKS and PRINTS [15]–[18].

It is worth noting that biological motifs fall into at least three somewhat distinct classes. The first comprises short motifs often found at functional sites of biopolymers, such as cleavage sites, binding sites and attachment sites. These short motifs probably arise through convergent evolution as often as not. The second comprises longer protein motifs associated with globular structural domains. These often, if not always, arise through divergent evolution. Finally, recurring motifs can arise from evolutionarily recent duplications, such as DNA transposons. It is not clear that these categories are best tackled by a single motif discovery method. glam2 is primarily aimed at short motifs for functional sites, although it performs respectably for the other categories.

In an ideal world, simple motifs would directly encode biological functions, as is the case with the triplet genetic code for amino acids for example. In reality, protein phosphorylation sites and the like may be encoded in a more complex and dispersed fashion, and in the worst case we would have to understand the full biophysics of the molecule in order to predict its function. Nevertheless, there is often at least a correlation between motifs and functional sites, which is useful. This is illustrated well by the ELM server, which uses protein motifs as a first step in predicting functional sites, and filters the predictions by criteria such as cell compartment and globular domain clash [15]. Thus, refining known motifs and discovering new motifs will be useful for identifying functional sites.

Most motif discovery algorithms are limited to gapless motifs. The main reason for this is that the motif discovery process becomes more difficult when gaps are allowed due to an explosion in the number of possible variations. Gapped motifs are ubiquitous in biology, however. Many of the motifs described in protein motif databases such as ELM and PROSITE contain variable length gaps. Transcription factor complexes can have DNA binding motifs with variable width spacers, and some DNA motif discovery algorithms are specialized to finding bipartite motifs – two motifs separated by a single, variable-length spacer.

There are some existing methods for discovering gapped motifs, but they do not appear to be widely used. pratt discovers gapped motifs, in the form of regular expressions, in protein sequences [19]. Regular expressions may have trouble capturing subtle motifs, because they specify exactly which residues and spacers are allowed at each position, and do not allow a better match in one part to compensate for a worse match in another part. Since they make such detailed specifications, it may also be hard to discover accurate regular expressions from small numbers of examples. In any case, glam2 re-discovers PROSITE motifs more sensitively than pratt (see below).

So-called profile hidden Markov models (HMMs) have been used to represent protein structural motifs with gaps, notably in the sam and hmmer packages, and HMM training algorithms can be used to discover such motifs [20]–[22]. It is telling that, while sam and hmmer are extremely successful and widely used, they are mainly used for motif scanning, and rarely for *ab initio* motif discovery. Recent versions of hmmer do not even retain the training algorithm. In fact, glam2 can be regarded as an HMM training method similar to these. A key difference is that, while sam and hmmer optimise the HMM parameters (the transition and emission probabilities), glam2 “integrates out” these parameters (Materials and Methods, Text S1), and directly optimises the motif alignment. One consequence is that glam2 can use a better-characterised heuristic to search for the globally optimum solution: simulated annealing, rather than expectation-maximization with noise injection. (Expectation-maximization alone is well-characterized, but it only finds local optima.) glam2 actually uses the same stochastic traceback step as hmmer, but since hmmer optimises the parameters rather than the alignment, this is not true simulated annealing, as pointed out by its author [22]. The yebis program also discovers gapped motifs, in DNA only, using an *ad hoc* HMM training method [23].

A dynasty of Gibbs sampling algorithms has been developed, which allow for gapped motifs with steadily increasing generality. The original Gibbs sampler only found ungapped motifs [24]. The second generation method allowed for discontiguous motifs, where poorly conserved positions within a motif are not considered part of the motif (“turned-off ”) [10]. This allows a limited form of insertion, which must be the same size in all motif instances. A successor program named probe is aimed at protein structural motifs, and it models a motif as multiple separated blocks, where each block may be discontiguous [25]. Most recently, Neuwald and Liu extended probe to allow general insertions and deletions within blocks, using an HMM very similar to the profile HMMs of sam and hmmer [26]. Since glam2 is also an extension of Gibbs sampling to allow general indels, it is somewhat similar to this method, but there are the following important differences:

- Neuwald and Liu use a more complex motif model, designed for protein structural motifs, and much more sophisticated alignment-editing operations and annealing schemes. However, some of their alignment-editing operations are awkward and violate the detailed balance condition of simulated annealing.
- The central step of re-aligning one sequence is carried out differently. glam2 uses the stochastic traceback algorithm to directly sample one alignment according to its score. Neuwald and Liu, in contrast, sample HMM transition and emission probabilities, then obtain the optimal alignment, and finally accept or reject this alignment in the standard Monte Carlo fashion.
- Neuwald and Liu use a simple Dirichlet prior for amino acid frequencies, which lacks information on their tendencies to align with one another, whereas glam2 uses Dirichlet mixtures, which can provide such information. Dirichlet mixtures will be more powerful for small numbers of sequences, but the simpler approach may be sufficient for large numbers of sequences. (glam2 can use either approach.)
- glam2 uses position-specific insertion and deletion probabilities, whereas Neuwald and Liu use universal insertion and deletion probabilities (within blocks). This is important because real motifs tend to concentrate insertions and deletions in a few positions.

This publication aims to make gapped motif discovery as powerful and ubiquitous as gapless motif discovery. We describe the glam2 algorithm for discovering gapped motifs, and a companion scanning algorithm, glam2scan. In the following, we first give an overview of glam2 and glam2scan, followed by more details on the methods. Full technical details are in Text S1. We then assess their performance at three different kinds of task: re-discovering PROSITE motifs, refining and then scanning ELM motifs, and aligning BAliBASE sequences. Finally, we give two examples of using these methods to discover kinase substrate motifs and to identify DNA target sites of the LIM-only complex. The results show that glam2 and glam2scan are very capable of identifying gapped motifs, especially short linear motifs.

## Materials and Methods

### Overview of glam2 and glam2scan

**Figure 1:**
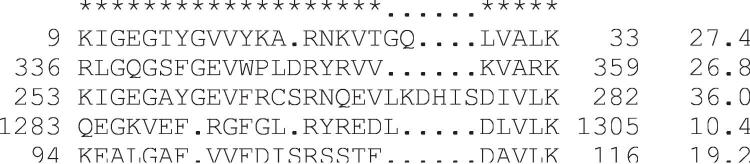
A typical motif alignment from glam2. The stars indicate the key positions. The residues inserted between key positions are not considered aligned to each other: their column placement is arbitrary. The numbers on either side of the aligned segments indicate the coordinates of each segment within the sequence. The decimal numbers on the right are the marginal scores of each aligned segment.

glam2 examines a set of sequences provided by the user, and returns an alignment of segments of these sequences. A typical alignment is shown in Figure 1. Each sequence contributes at most one segment to the alignment. Our approach assumes that a motif is defined by residue preferences at certain positions, which we call key positions. These are analogous to the “turned-on” columns of the second-generation Gibbs sampler, or to the match states of a profile HMM. In a particular motif instance, some key positions may be deleted, and residues may be inserted between key positions (Figure 1).

glam2 defines a scoring scheme for alignments such as that in Figure 1. It rewards alignment of identical or similar residues in the same key position, and penalizes deletions and insertions. However, deletions and insertions are penalized less strongly if they repeatedly occur in the same locations. This is reasonable because some locations in a motif may be more prone to deletions or insertions than others. Having defined a scoring scheme for alignments, it is straightforward to calculate the marginal score of one aligned segment: the score of the alignment including this segment minus the score of the alignment excluding this segment. These marginal scores reflect how well each segment matches the other segments.

Having defined a scoring scheme, glam2 attempts to find a motif alignment with maximum score. Even in the gapless case, the number of possible alignments is too huge to enumerate, and there is no practical algorithm to guarantee finding the optimal alignment. This problem is only exacerbated in the gapped case. Thus glam2 uses a heuristic optimisation method – simulated annealing – highly analogous to the optimisation methods of the gapless Gibbs samplers [10],[27].

Simulated annealing takes an initial, presumably non-optimal, alignment and repeatedly makes changes to it. These changes have an element of randomness: they generally increase the score, but sometimes decrease it, which avoids getting stuck in local optima. The process is analogous to crystallization in a cooling material. Two types of change are performed by glam2, which we call site sampling and column sampling, because they are analogous to similarly-named procedures in the original Gibbs sampler [10],[24]. Site sampling adjusts the alignment of one sequence to the motif, using the clever stochastic traceback procedure from hmmer to efficiently sample one from all possible such alignments [22]. In column sampling, one key position is moved, added, or deleted. These changes are carefully designed to satisfy the reversibility and detailed balance conditions of simulated annealing (Text S1). Such changes are applied until the score fails to improve for *n* (e.g. 10000) changes in succession. To check that a reproducible, high-scoring motif has been found, the whole procedure is repeated *r* (e.g. 10) times from different random starting alignments.

glam2's behaviour can be controlled with numerous adjustable parameters. The allowed alignments can be constrained by specifying a minimum number of key positions (*a*), a maximum number of key positions (*b*), and a minimum number of segments in the alignment (*z*). This *z* parameter is a useful generalization of the OOPS (one occurrence per sequence) and ZOOPS (zero or one occurrence per sequence) modes of previous motif discovery algorithms [28]. The annealing follows a simple geometric cooling schedule with initial temperature *t* and cooling rate *c* per *n* changes. glam2 can find the optimal number of key positions more quickly if the initial number (*w*) is set to a near-optimal value. All parameters have sensible default values.

glam2scan takes a motif found by glam2, and scans it against a database of sequences. It performs short-in-long alignments of the motif against the sequences, using position-specific residue scores, deletion scores, and insertion scores, which are derived from the glam2 alignment. The highest-scoring such alignments are reported.

### The glam2 Scoring Scheme

glam2's formula for assigning scores to alignments is a generalization of the formula used by previous Gibbs samplers for alignments without indels [27],[29]. Previous Gibbs samplers have used a log likelihood ratio formula:
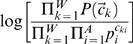



Here, *W* is the width of the alignment, *A* is the alphabet size, *p_i_* is the abundance of the *i* ^th^ residue type, *c_ki_* is the count of the *i* ^th^ residue type in the *k* ^th^ column of the alignment, and 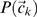 is the probability of observing the count vector 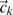 in an aligned column. 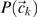 is given by the following formula (dropping the *k*):
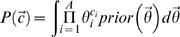



Here, 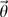 is a vector of residue probabilities, and the integral is over all possible values of this vector. Previous Gibbs samplers have used a Dirichlet distribution for 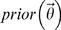, whereas glam2 uses a Dirichlet mixture. Dirichlet mixtures are explained in, for instance, [30].

glam2, in addition, allows deletions and insertions in the alignment. The numerator in the log likelihood ratio formula now becomes:
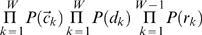



Here, *d_k_* is the number of deletions in the *k* ^th^ column (key position) of the alignment, and *r_k_* is the number of inserted residues (in all sequences) between columns (key positions) *k* and *k*+1. *P*(*d_k_*) and *P*(*r_k_*) are given by these formulas (dropping the *k*):
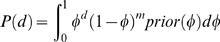


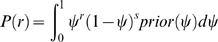



Here, *m* is the number of non-deleted residues and *s* is the total number of sequences, so that *d*+*m* = *s*. glam2 uses Beta distributions, which are a type of Dirichlet distribution, for *prior*(*φ*) and *prior*(*ψ*). Thus, the scoring scheme for deletions and insertions is entirely analogous to that for aligned residues. For full details, see Text S1.

### Site Sampling

In site sampling, one of the input sequences is chosen at random, removed from the alignment (if it is present in the alignment), and then re-aligned to the motif. All possible alignments of substrings of this sequence to the motif are considered. One alignment is chosen at random, with probability proportional to the resulting alignment's likelihood ratio, as defined above, raised to the power of 1/*t* (“heated”). This scheme satisfies the criteria for simulated annealing.

The re-alignment is accomplished by dynamic programming followed by a stochastic traceback ([22], Text S1). Briefly, the dynamic programming step calculates a matrix of values *M*(*i*,*j*) equal to the sum of the heated likelihood ratios of all alignments ending at the *i* ^th^ key position in the motif and the *j* ^th^ residue in the sequence. This is similar to standard dynamic programming algorithms for finding optimal alignments, except that maximization is replaced by summation. The stochastic traceback step is also similar to the standard traceback used to find optimal alignments, except that it chooses a random path through the matrix, weighted by the *M*(*i*,*j*) values, rather than taking the optimal path.

### Column Sampling

The site sampling moves of the original gapless Gibbs sampler were prone to getting stuck in shifted versions of the optimal motif [24], and glam2 has an analogous problem. Column sampling overcomes this problem, and in addition, allows the number of key positions in the motif to be adjusted.

In column sampling, one key position is chosen at random, and removed from the alignment. This means that the residues that were in this key position now become regarded as insertions between the preceding and following key positions. Then, a new key position is added to the alignment. Several ways of adding a key position are considered, and one of these is chosen at random, with probability proportional to the resulting alignment's likelihood ratio, as defined above, raised to the power of 1/*t*.

So far, this is highly analogous to site sampling. However, the number of ways of adding a key position to a gapped alignment is generally astronomical, and we do not have a clever algorithm to consider them all efficiently, so we must consider a subset. Furthermore, this subset must include the possibility of returning to the original alignment by adding back the key position that was removed, in order to satisfy the reversibility requirement of simulated annealing. Thus, we consider all ways of adding a key position that preserve certain properties of the key position that was removed (Text S1).

Finally, we allow the number of key positions to increase by sometimes neglecting to remove the chosen key position, and we allow the number of key positions to decrease by sometimes neglecting to add a new key position. The probabilities of not removing and not adding a key position are carefully chosen to satisfy the detailed balance condition of simulated annealing: the details are interesting but somewhat technical (Text S1).

### The Initial Alignment for glam2

The simulated annealing procedure for finding high-scoring alignments needs to start from some initial alignment. The initial alignment for glam2 is constructed as follows. The number of key positions (aligned columns) is set to a fixed value, *w*, chosen by the user, by default 20. Starting with an empty “alignment” containing zero sequences, the input sequences are taken one-by-one, in random order, and added to the alignment using a site sampling move with temperature *t* = 1. Ideally, the initial alignment should have no effect on the result, since simulated annealing finds the globally optimal alignment. In practice, the *w* parameter does influence the result, though this influence decreases as the annealing is allowed to run for longer.

### Optimising glam2 Parameters

The glam2 algorithm involves many adjustable parameters, and we wish to find suitable parameter settings for effective motif discovery. It is likely that different settings will be optimal for different scenarios (e.g. protein versus DNA motifs, many short input sequences versus few long input sequences), and we cannot deal with all conceivable scenarios here.

The glam2 parameters fall into two categories: those that affect the scoring scheme for motif alignments, and those that affect the search algorithm to find high-scoring alignments. Of these, the former are more fundamental, since we must be able to recognise good alignments before we can contemplate searching for them. The score parameters are further divisible into those that determine scores for aligned residues, those that determine scores for deletions, and those that determine scores for insertions. Aligned residue scores are determined by a Dirichlet mixture, which is non-trivial to optimise, and we use parameters derived in previous work: for proteins we use recode3.20comp from sam, and for DNA we use a single Dirichlet component with all pseudocounts = 0.4 [27],[30]. Deletion and insertion scores are each determined by a Beta distribution, which has only two pseudocount parameters. It is straightforward to find pseudocount values that best fit a given set of typical alignments (Text S1), but it is not so obvious whence to obtain such alignments.

We reasoned that, if we use glam2 with sensible guesses for these pseudocount parameters, we will obtain fairly good alignments, and these alignments can then be used to fit the pseudocounts. This procedure can be iterated until the fitted values stop changing. We took this approach with 58 PROSITE alignments and separately with 141 BAliBASE alignments (see Results). The alignments are, in fact, fairly accurate (see Results), and in both cases the following parameter settings are close to optimal. Pseudocounts for deletions: *D* = 0.1, *E* = 2. Pseudocounts for insertions: *I* = 0.02, and *J* = 1. All results reported here use these settings. Since these settings were tuned on protein alignments, they may not be ideal for DNA alignments.

The main parameters that affect the search algorithm are *r*, *n*, *t*, *c*, and *w*. The initial width (*w*) is important but obviously problem-specific: it helps to specify a good estimate of the true motif width. The other parameters were selected based on experiments with glam [27], and additional *ad hoc* experimentation. There is likely scope for improved annealing procedures such as simulated tempering [31]. None of the parameters were optimised based on performance on the assessments described here.

### glam2scan

glam2scan uses standard methods to search for motif instances in a sequence database. Each sequence is scanned in turn, using the Waterman-Eggert algorithm to find multiple motif hits per sequence [32]. The top *n* hits in the whole database, where *n* is a parameter chosen by the user, are collected using a heap, which is a standard data structure. For full details, see Text S1.

### Program Parameters for PROSITE

The programs were run with the following options. glam2: -z 10,000 (force all sequences to participate), -b 10000 (effectively no upper limit on motif width), -n 100000 (slow and thorough). sam-t2k: -homologs -tuneup. Note that glam2 and sam-t2k use the same Dirichlet mixture prior (recode3.20comp). pratt: default options. Unlike glam2 and sam-t2k, pratt sometimes returns multiple motif hits per sequence (in 19 out of 58 cases): to deal with this, pratt alignments were constructed only from sequences with one hit. This harms pratt's sensitivity, but the main conclusion is not in jeopardy, because at most 19 cases are affected whereas glam2 has higher sensitivity in 56 out of 58 cases (Table 1).

**Table 1:** Comparison of glam2 with sam-t2k and pratt on 58 PROSITE motifs.

Comparison	Sensitivity			PPV		
Method	glam2 Better	glam2 Worse	*P*	glam2 Better	glam2 Worse	*P*
sam-t2k	42	4	5.1e-09	51	6	5.7e-10
pratt	56	0	2.8e-17	30	27	0.79

The glam2 Better columns indicate the number of cases, out of 58, where glam2 has a higher value (of sensitivity or PPV) than sam-t2k or pratt. The glam2 Worse columns indicate the number of cases where glam2 has a lower value. The *P* columns indicate the probability of this difference or greater arising by chance (two-sided binomial test).

### Program Parameters for ELM

The glam2 parameters used are: -z 10,000 (force each sequence to contribute one site) and -n 100,000 (slow and thorough). A minority of the ELM REs are anchored at the N-terminus (C-terminus) of the protein: in these cases, we ignored glam2scan hits outside of the first (last) 20 residues.

### Program Parameters for BAliBASE

glam2 was run with the following options: -z 10,000 (force all sequences to participate), -b 10,000 (effectively no upper limit on motif width), and -n 100,000 (slow and thorough). In addition, the initial motif width (-w) was set to the length of the shortest sequence in the set being aligned. Finally, we used non-default annealing options -t 1.5 and -c 2.25. The default annealing options produce slightly worse results for the category “cases with divergent subfamilies”, and very similar results for all other categories. We suspect that the sub-families in this category give rise to strong local optima, and the higher initial temperature may help to escape these.

### Gene Names and Accession Numbers

The UniGene names and Refseq RNA accession numbers (in parentheses) for the genes mentioned in this paper are: Lmo2 (NM_008505), Tal1 (NM_011527), Gata1 (NM_008089), E2a/Tcfe2a (NM_011548), Ldb1 (NM_010697), Tgfb1 (NM_011577), Klf13 (NM_021366), Gata5 (NM_008093), P4.2 (NM_013513), Gypa (NM_010369) and Cdh5 (NM_009868).

## Results

### Rediscovering PROSITE Motifs

We wished to assess glam2's efficacy by using it to re-discover known motifs. For this purpose, we used the PROSITE database (release 19.25 of 18-Apr-2006). PROSITE is a database of protein motifs represented by either “patterns” (regular expressions) or “profiles” (hidden Markov models) [16]. To test glam2, we extracted all variable-length patterns (since these entail indels), and obtained the sequences annotated in PROSITE as true positive hits to each pattern.

Since glam2 produces motif alignments, we desired a set of gold standard alignments to compare them to. To construct gold standard alignments, we used ps_scan to locate the motifs in the sequences, and lined up equivalent residues in the ps_scan hits [33]. Sequences not having exactly one ps_scan hit were discarded. Finally, we removed highly similar sequences from each set using blastclust -L 0 -S 0 (ftp://ftp.ncbi.nlm.nih.gov/blast/). This step is important because, if highly similar sequences are present, glam2 may, not unreasonably, detect this extended similarity rather than the desired motif. Sets with fewer than three remaining sequences were discarded. These steps resulted in 58 test sets with a total of 368 sequences (Dataset S1).

For this assessment, it is necessary to measure the similarity of a predicted motif alignment to a gold standard motif alignment. Our primary measure is sensitivity of aligned residue pairs: the number of correctly aligned residue pairs as a percentage of the total number of aligned residue pairs in the gold standard. We also measured the positive predictive value (PPV): the number of correctly aligned residue pairs as a percentage of the total number of aligned residue pairs in the prediction.

In this study, sensitivity is more informative than PPV, for two reasons. Firstly, unlike many other prediction assessments, there is no trivial way to achieve 100% sensitivity, because it is not possible to align all residue pairs at once. Thus, 100% sensitivity is significant and potentially useful, regardless of the PPV. Secondly, the PROSITE patterns probably err towards minimality, excluding subtle similarities that are hard to represent with regular expressions. Thus, excess aligned residues in the prediction are more likely to be biologically correct than are missing aligned residues.

We wished to compare glam2 to other tools that could be used to discover these motifs. Most motif discovery programs cannot handle variable-length motifs at all, and thus are ruled out. The first tool we compared against is sam-t2k (from sam version 3.5), which can discover motifs by fitting hidden Markov models [21]. Since sam-t2k was not designed to find short motifs, we might expect it to return large alignments with low PPV – we include the sam-t2k comparison to highlight the paucity of methods that are suited to this task. We also compared against pratt (version 2.1), which discovers motifs in the form of regular expressions [19]. Since the test cases are derived from regular expression motifs, this assessment may be biased in favour of pratt.

**Figure 2:**
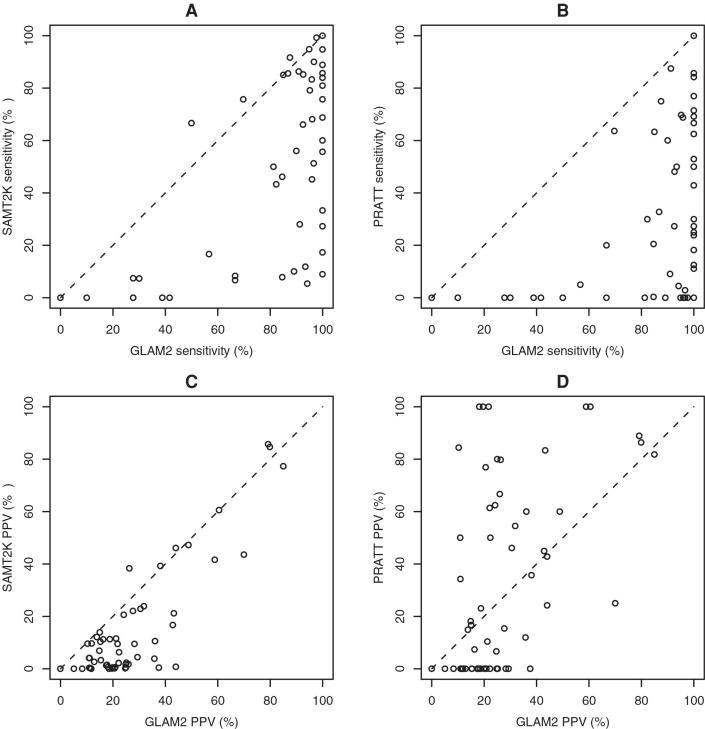
Sensitivity and positive predictive value of glam2 compared to sam-t2k and pratt on 58 PROSITE motifs.

The sensitivity and PPV of glam2 on each of the 58 test cases, compared to sam-t2k and pratt, is shown in Figure 2. glam2 is generally the most sensitive method, often achieving 100% sensitivity or close to it. Interestingly, glam2 and sam-t2k often find considerably more extended alignments than the gold standard motifs, with low PPV. This suggests that either many of the motifs have large, subtle extensions not recorded in PROSITE, or many datasets have evolutionary or structural relations subtle enough to survive blastclust. In the latter case, it is not clear whether the smaller motif exists independently of the more extended similarity. pratt often achieves much higher PPV than the other methods, no doubt because it uses the same regular expression model as PROSITE, which does not capture the more subtle similarities. The increase in sensitivity of glam2 over the other two methods is statistically significant, as is its PPV compared to sam-t2k's (Table 1).

### Refining ELM Motifs

**Figure 3:**
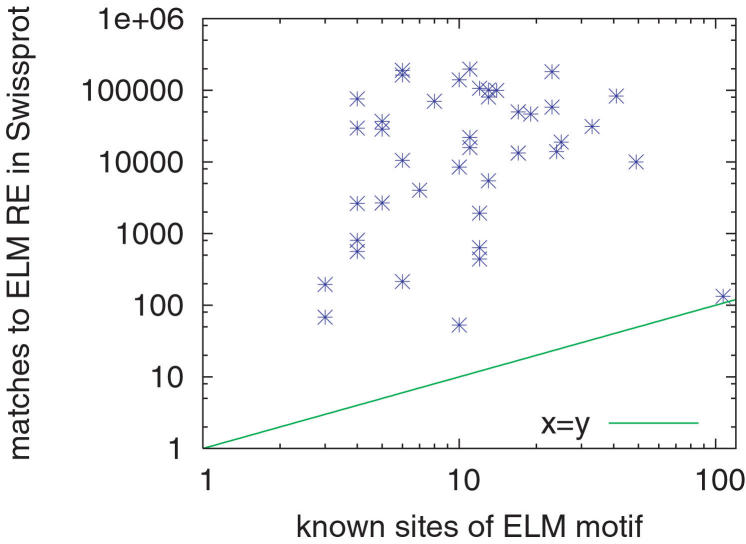
Non-specificity of ELM motif regular expressions. Each point represents one of the 41 ELM motifs used in this study. The *x*-value of the point is the number of known sites, and *y* gives the number of predicted sites in Swiss-Prot sequences.

The Eukaryotic Linear Motif (ELM) resource [15] is a database containing 115 linear motif regular expressions (REs) corresponding to protein functional signals such as binding, interaction and protease cleavage sites. Many of the ELM motifs are annotated with lists of known sites in sequences in the Swiss-Prot [34] database. ELM motifs tend to be fairly short and non-specific; some contain as few as two specified amino acids. For example, the motif for the site for attachment of a mannosyl residue to a tryptophan is “W‥W” (ELM entry “MOD_CMANNOS”). As a result, searches using ELM regular expression motifs are subject to making large numbers of false positive predictions. This problem is illustrated in Figure 3, which plots the number of matches to the ELM regular expression in Swiss-Prot against the number of annotated sites for the 41 ELM motifs used in this study. It shows clearly that many ELM motifs are extremely non-specific, matching orders of magnitude more positions in Swiss-Prot sequences than are annotated as known sites.

It would be useful to be able to use glam2 to produce more specific models of linear motifs than the regular expressions (REs) available in the ELM database. We do not consider sam-t2k here, since it is not designed to find short motifs, and in practice glam2 finds short motifs more accurately (see above). The idea is to use the known sites for an ELM motif, with some flanking sequence, as input to glam2, to discover a glam2 motif. Then, we use glam2scan to search novel protein sequences for matches to the motif. In order to evaluate the benefits of this approach, we need a way to estimate the accuracy of ELM and glam2 motifs. As our figure of merit, we chose to use “FP_N”, the number of false positive predictions at a sensitivity of *N*%. To estimate the FP_N of an ELM regular expression motif, we use *N*% of the difference between the number of matches (*H*) to the RE in the Swiss-Prot database and the number of known sites (*K*) for the motif. This is reasonable since we expect there to be (*H*−*K*)*N*/100 false positives in any randomly chosen *N*% of the matches to the RE.

To measure the accuracy of glam2 motifs derived from sites annotated in the ELM database, we first create sequence sets containing the full-length proteins annotated as containing known sites for each ELM RE. In order to avoid biasing the motifs discovered by glam2, we purge the sequence set using the purge [10] program so that no two sequences have BLOSUM-62 score greater than 150. Sets with fewer than three sequences after purging are discarded. We then extract the sites along with ten flanking residues on each side to create 41 sets of extended sites (Dataset S2). These are input to glam2, producing 41 motifs (Dataset S3). Each motif discovered by glam2 is used to search the Swiss-Prot sequence database via glam2scan. For each known site, we count the number of false positive sites (FP) with better glam2scan scores. Our estimate of FP_N for glam2 motifs is the *N* ^th^ percentile of these FP values. For example, if there are 100 known sites for a motif, the FP_N value would be the *N* ^th^ *smallest* observed FP value.

In a separate, more stringent measure of the accuracy of glam2 motifs, we perform leave-one-out cross-validation (CV) on the set of known sites, and count the number of false positives (FP) with better glam2scan scores than the left-out site in a scan of Swiss-Prot. Our CV estimate of FP_N for glam2 motifs is the *N* ^th^ percentile of the FP values observed during CV. Note that we did not perform any cross-validation on the ELM REs since this is impossible because they were manually generated by the curators of ELM. This puts glam2 at a disadvantage in a comparison such as ours, since the curators of ELM could optimize their REs on all the known sites, whereas glam2 is always tested on sites that it has not seen. This disadvantage is likely to be especially pronounced when measuring FP_100, because the ELM REs are fitted to all the unusual edge cases, which are hardest in cross-validation tests.

**Figure 4:**
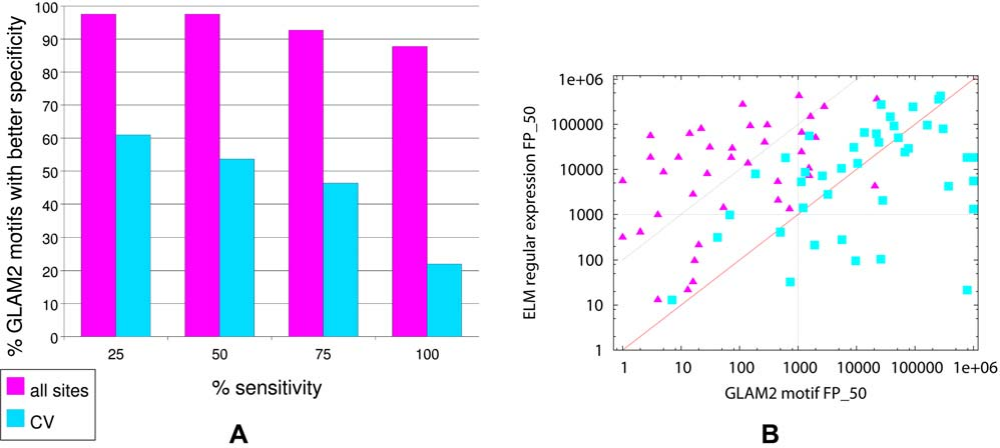
Sensitivity versus specificity trade-off of glam2 motifs. (A) shows how often the glam2 motif has better specificity than the corresponding ELM RE as a function of the sensitivity level. (B) shows the specificity (FP_50) of the ELM RE and the glam2 motif for each of the 41 ELM entries studied here. Each point represents one ELM motif, with x and y giving the the FP_50 of the glam2 motif and of the ELM RE, respectively. Triangles are motifs learned from all sites; squares show cross-validated results.

glam2 motifs provide a good way to improve the specificity of ELM REs, as is evident in Figure 4A. For example, at sensitivities up to 50%, the glam2 motif learned from all the ELM sites is more specific than the corresponding ELM RE in 98% (40 out of 41) cases. Even at a sensitivity level of 100%, the glam2 motif is more specific in 88% (36 out of 41) of the cases tested. In the cross-validated test, which severely penalizes glam2, glam2 motifs are more specific than ELM REs at sensitivity levels below 75%. The improved specificity of the glam2 motifs is made more apparent in Figure 4B. At a sensitivity level of 50%, glam2 motifs learned from all of the known sites tend to be orders of magnitude more specific. In about half the cases, the ELM RE has more than 100 times more false positives than the glam2 motif (triangles above the upper diagonal line in Figure 4B). In only one case is the glam2 motif less specific than the corresponding ELM motif. The cross-validated glam2 motifs are, on average about as specific as the ELM REs (squares in Figure 4B). The five outliers (square points along the right border of the plot) are motifs with only three or four sites (after purging). This means that glam2 was only given two or three sites from which to learn the motif during each cross-validation run, an extremely difficult task. For motifs with more than four sites, the cross-validation study shows that glam2 motifs generalize about as well as the ELM REs.

Some of the ELM REs have more than an order of magnitude more false positives than the corresponding cross-validated glam2 motif (Table 2). In one case, the ELM RE predicts nearly 8000 false positives (FP_50 = 7964), whereas the glam2 motif predicts only 187. In five out of 41 cases, the glam2 motif has an FP rate more than ten times smaller than the ELM RE.

**Table 2:** ELM families where glam2 motifs are massively more specific.

ELM Family	Specificity (FP_50)		Improvement (fold)	ELM RE
	glam2	ELM RE		
LIG_CtBP	611	18120	29.7	[PG][LVIPME][DENS]L[VASTRGE]
LIG_CYCLIN_1	26344	275157	10.4	[RK].L.{0,1}[FYLIVMP]
MOD_CMANNOS	187	7964	42.6	W‥W
MOD_TYR_ITAM	68	975	14.3	[DE]‥(Y)‥[LI].{6,12}(Y)‥[LI]
MOD_TYR_ITIM	1554	55415	35.7	[ILV].(Y)‥[ILV]

Improvement in specificity is defined as (ELM FP_50)/(glam2 FP_50).

### Aligning BAliBASE Sequences

Since glam2 discovers and aligns motifs allowing arbitrary indels, it is effectively a multiple sequence alignment tool, bridging the traditionally separate domains of motif discovery and multiple alignment. Thus, we wished to assess glam2's efficacy in typical multiple alignment scenarios. For this purpose, we used the BAliBASE multiple alignment benchmark [35]. We used the older BAliBASE 2.01 rather than the newer BAliBASE 3, simply because more multiple alignment tools have been tested on BAliBASE 2.01, facilitating comparisons.

BAliBASE includes 141 protein alignments in five categories: (1) equidistant sequences, (2) cases with one highly divergent sequence, (3) cases with divergent sub-families, (4) alignments with N- and C-terminal extensions, and (5) alignments with large internal insertions. The alignments are based on three-dimensional structural superpositions, so they can be regarded as structural motifs, rather than the shorter functional site motifs that glam2 is primarily designed for. Each alignment is annotated with “core blocks”, indicating the columns that are thought to be reliably aligned. All categories except (4) use partial sequences trimmed to the alignable region, which is unrealistically favourable to global alignment algorithms, as has been noted by others [36],[37]. Conversely, category (4) is the most motif-like, and so we might expect glam2 to excel on this one.

The accuracy of glam2's alignments was measured using the bali_score program included with BAliBASE, which reports two statistics for each alignment: SP and TC. SP is the number of correctly aligned residue pairs as a percentage of the total number of aligned residue pairs in the BAliBASE alignment. (It is the same as the sensitivity measure used in the PROSITE assessment above.) Only residues in BAliBASE core blocks were counted. TC is the number of correctly aligned columns as a percentage of the total number of columns in BAliBASE core blocks.

The average SP and TC scores for each BAliBASE category are shown in Table 3. These results are directly comparable to those in Table 1 of [37], Table 1 of [38], and Tables 2 and 3 of [39], which collectively give results for these alignment tools: Align-m, ClustalW, Dialign, Kalign, MAFFT, MUSCLE, ProbCons, and T-Coffee. For the motif-like category (4), glam2 achieves slightly better results than all other tools. Since there are only twelve alignments in this category, this result is promising but not conclusive. For the other categories, glam2 achieves comparable results to the other tools, but it is not the best method.

**Table 3:** Average glam2 performance on each BAliBASE category.

Category	1	2	3	4	5
Alignments	82	23	12	12	12
Average SP	83.3 (76.6–90.1)	92.1 (88.4–94.4)	72.0 (68.4–84.3)	94.4 (79.3–93.8)	91.6 (85.9–98.1)
Average TC	77.5 (70.9–82.6)	55.7 (35.9–61.3)	45.0 (34.4–61.3)	81.1 (45.1–81.0)	77.3 (63.8–92.2)

The first row indicates the number of alignments in each category. The numbers in parentheses are the lowest and highest values observed in previous tests involving eight methods [37]–[39]. Note that no single method produces all of the highest values.

### Discovering Motifs in Protein Kinase Substrates

Enzymes of the eukaryotic protein kinase superfamily are ubiquitous in nature and are involved with the regulation of essentially every cellular process [40]. These protein kinases phosphorylate substrate proteins at either serine/threonine or tyrosine residues. To ensure signaling fidelity, a protein kinase acts on a discrete set of substrates. Two major factors determine how protein kinases recognise their substrates [41]. The first, termed peptide specificity, describes the interaction between a binding pocket in the protein kinase catalytic domain and the substrate residues either side of the phosphorylated residue. The second factor, termed substrate recruitment, describes any additional process that facilitates formation of the protein kinase-substrate complex. Substrate recruitment is often mediated through docking interactions between a binding site on the protein kinase and a short peptide motif on the substrate [42]. Elucidation of these motifs may provide us with a code for cellular signaling.

Protein kinase substrate sequences were obtained from the phospho.ELM database [43], and grouped by kinase family. Redundant sequences were removed from each group using purge (BLOSUM-62 score cutoff = 150). Groups with 3 or more remaining sequences (Dataset S4) were submitted to glam2, with parameters -a 3 (minimum width), -b 7 (maximum width), -w 5 (initial width), and -n 100000 (slow and thorough). These width parameters are based on the sizes of known phosphorylation and docking motifs in substrates [42]. Results were compared to known motifs using PhosphoMotif Finder and ELM [15],[44].

glam2 identified a number of interesting motifs in substrates of both tyrosine and serine-threonine protein kinases (Table 4). The motifs include both putative phosphorylation sites (e.g. a GSK3 kinase site in substrates of Akt kinase) and domain binding sites. glam2 was particularly effective at identifying proline-rich regions, finding putative motifs for SH3 domain-binding, WW domain-binding and proline-directed kinase phosphorylation. Strikingly, all of the sequences in each group participated in the motif alignments, even though we did not force this to happen (with glam2's *z* parameter), suggesting that glam2 is finding real, shared motifs.

**Table 4:** glam2 motifs in protein kinase substrates.

Kinase (# substrates)	glam2 consensus motif	Known motif	Known annotation
Tyrosine kinases			
Abl (10)	PPPPPPA	X{3}[PV]X{2}P	SH3 domain-binding
Src (14)	ELPPLPP	X{3}[PV]X{2}P	SH3 domain-binding
Serine-threonine kinases			
Akt/Rac (3)	SRLRSCT	X{3}([ST])X{3}[ST]	GSK3 kinase substrate
CaMK-III (4)	EEEARE	X[DE]X[ILV]	PDZ domain-binding
CDC15 (4)	PSNPPPS	X{3}[PV]X{2}P	SH3 domain-binding
CDK (55)	DEE.....EEEE	-	-
CDK2 (7)	EEDD	-	-
CDK5 (7)	EEEEEDD	-	-
ERK-II (8)	PSSPRQE	X{3}([ST])PX	WW domain, Pro-directed kinase
		X{3}[PV]X{2}P	SH3 domain-binding
ERK/MAPK (28)	PSPPPG	X{3}([ST])PX	WW domain, Pro-directed kinase
		X{3}[PV]X{2}P	SH3 domain-binding
GSK3-II (7)	DDDEDEE	-	-
Polo (9)	EEEGEE	-	-

In some cases the glam2 alignment matched a known motif in some, but not all substrate sequences. This is the case for CaMK-III (calmodulin-dependent kinase) substrates (4 sequences), where two sequences contained a consensus PDZ domain-binding motif X[DE]X[ILV] and the other two sequences differed by having Ala at the [ILV] position. This suggests that (i) the results from glam2 are meaningful for some, but not all sequences in a group of substrates, (ii) the motif defined by glam2 is a genuine novel motif but resembles a known motif or (iii) the existing consensus for some known motifs could be redefined on the basis of the glam2 motif. Examples of complex formation involving PDZ domains and other calmodulin-dependent kinases have been reported [45].

glam2 also identified high-scoring motifs in several groups of substrate sequences to which function could not be assigned. Of particular note is the presence of short sequences with a high proportion of aspartate and glutamate residues in substrates of CDK-type and Polo kinases (Table 4). Evidence of a biological function for these motifs was not found in existing motif databases or a literature survey. However, their conservation in substrates of related kinase families strongly implies a role in kinase-substrate interaction.

### Exploring Transcriptional Regulation with glam2 and glam2scan

In this section we investigated the utility of glam2 and glam2scan for studying transcriptional regulation. Because glam2 motifs can model transcription factor binding sites containing variable length spacers, we focused on a regulatory binding complex known to have sites of variable length. In particular, we used glam2 to discover a model of a bipartite DNA-binding motif associated with an erythroid protein complex centered on Lmo2 [46], and then used glam2scan to identify possible binding sites (and target genes) of this complex in the mouse genome. We then compared the predicted targets with the list of genes shown to be up-regulated by Gata-1 (one putative member of the Lmo2 protein complex) in a ChIP-chip study by [47].

**Figure 5:**
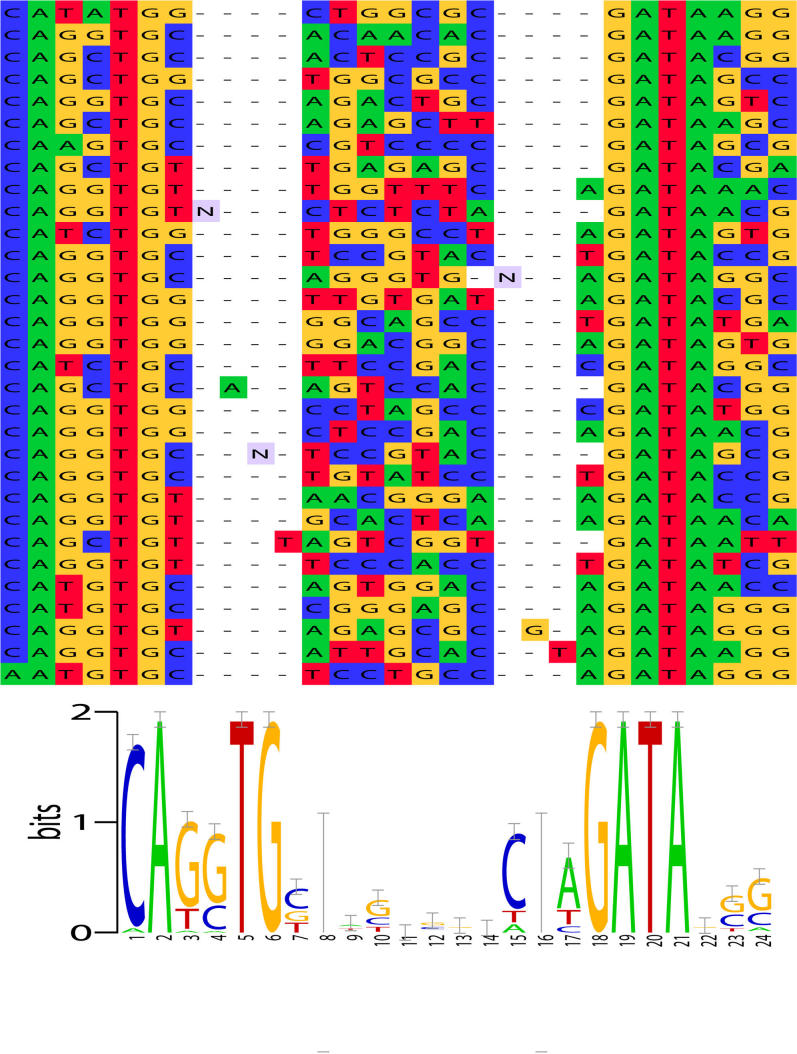
glam2 output on 31 clones that bind the Lmo2 complex. glam2 was run using default parameters on the clones identified in Figure 1A of [46]. The glam2 alignment is shown on the top, and the information content “LOGO” corresponding to the alignment is shown on the bottom. The glam2 alignment was pretty-printed using PFAAT [60]. The LOGO is corrected for small-sample size [61].

The Lmo2 complex consists of transcription factors E2a, Tal1 and Gata-1 bound to Lmo2 and Ldb1. A bipartite binding motif consisting of an E-box (CANNTG) and a GATA, separated by a spacer of length 8 to 10 basepairs, was previously determined using CAST-ing [46]. When given as input the 31 random DNA oligomers that bound this complex in the CAST-ing experiment, glam2 discovers this motif. The average length of the oligomers is about 31 bp, and glam2 is run with its default parameters. The alignment determined by glam2 is shown in Figure 5. glam2 exactly identifies the boundary of the E-box on the left and extends the GATA motif on right by three columns. glam2 correctly determines the need for up to two insertions to account for the variable (8–10 bp) spacer between the DNA regions bound by Tal1/E2a and Gata-1.

Using the glam2 alignment, glam2scan detects significantly high-scoring matches adjacent to the promoters of several important genes involved in erythropoeisis. Among these genes are the four known targets of the Lmo2 complex studied here,VE-cadherin (Cdh5) [48], P4.2 (Epb4.2) [49], glycophorin A (Gypa) [50] and complex member Gata-1 [51]. The high-scoring matches to the binding motif of the Lmo2 complex also include several that have not been previously reported to the best of our knowledge. In the 1 kb upstream regions of all mouse genes (downloaded from the UCSC genome browser http://hgdownload.cse.ucsc.edu/goldenPath/mm8/bigZips/), glam2scan detects a very strong match to transforming growth factor beta 1 (Tgfb1). The score of this match ranks 2 out of 17254 upstream regions. Tgfb1 is known to be involved in IL-3-dependent early erythropoiesis [52]. Strong matches are also seen for Klf1 (rank 42/17254), a very important transcriptional regulator of erythropoeisis, and for Gata-5 (rank 73/17254), which, like Gata-1, binds GATA DNA sites. These genes were not reported as having binding sites adjacent to their promoters by [46]. glam2scan finds moderate to weak matches in the 1 kb upstream regions of some the members of the Lmo2 complex–Lmo2 (rank 2327/17254), E2a (Tcfe2a, rank 1031/17254), Gata-1 (rank 1716/17254) and Ldb1 (rank 2812/17254).

The known binding site upstream of Gata-1 is outside of the 1 kb region, but the known site ranks 761/17254 when we scan the 2 kb regions of all mouse genes (2 kb regions downloaded from same source as 1 kb regions). The known site for P4.2 likewise has rank 287/17254 in the scan of the 2 kb upstream regions. The other two known sites are within 1 kb of the start of transcription of the glycophorin A and VE-cadherin genes, and glam2scan detects them with rank 386 and 429 out of 17254, respectively. The probability of getting the right promoter within the top 386 by chance alone is 0.0224.

We examined the fifty top-scoring genes using GOStat [53], looking for groups of genes that share common GO terms [54]. The most statistically significant GO terms shared by subsets of these fifty genes included hemopoeisis, shared by four genes (*p*-value 0.00156, false discovery rate 0.0902): Nkx2-3, Klf1, Tgfb1 and Il7, and cell differentiation, shared by twelve genes (*p*-value 0.000452, false discovery rate 0.0902): Nkx2-3, Kcnip3, Klf1, Stk4, Dazap1, Prop1, Gprin1, Nanos2, Cidea, Barhl2, Tgfb1 and Il7. Although a false discovery rate of 0.0902 is not highly significant, it is very encouraging that four of the fifty top-scoring genes detected by glam2scan are implicated in hemopoeisis, a process for which Lmo2 is now known to be essential [55].

Since the Lmo2-complex contains Gata-1, we looked at the response of Lmo2 transcription to the presence of nuclear Gata-1 reported in a previous study [47]. Lmo2 shows approximately 1.4-fold up-regulation (rank 1775 out of 5053 genes) at 3 hours after introduction of Gata-1 to the nucleus in an experimental system based on erythrocytes. (Expression data was downloaded from http://stokes.chop.edu/web/weiss/G1Eindex.html.) In this same study, the most highly up-regulated gene after 3 hours was Csf2rb1 (19.2-fold), and there is a high-scoring match to the glam2 alignment in the 2 kb upstream region of Csf2rb1. Its rank is 148 out of 17254 regions.

### Computational Requirements

glam2 is quite time-consuming in general, although it can be fast in favourable cases. For a small dataset (e.g. ten sequences of 100 residues each) with a strong motif, it can find a probably optimal alignment in seconds or tens of seconds on a standard computer. For slightly larger datasets and weaker motifs, it typically takes minutes or tens of minutes. To process many datasets, it becomes desirable to run them in parallel on a multi-CPU cluster. Proteins of typical length are processed several times more slowly than same-length nucleotide sequences, because glam2 uses a more complex Dirichlet mixture for proteins by default, and the Dirichlet calculations become the bottleneck. The time scales linearly with sequence length (assuming the motif width is bounded), making it difficult to analyse sequences longer than a few thousand residues, and impractical to analyse sequences much above ten thousand. Fortunately, just about all known proteins are under this limit. On the other hand, glam2 has modest memory requirements, and runs robustly without crashing.

glam2's behaviour with large numbers of sequences is more complex. The speed is not directly affected, and it can happily process ten thousand or more sequences, but the result will probably be far from optimal unless the annealing is slowed down by increasing the *n* parameter. Furthermore, column sampling becomes ineffective with large numbers of sequences, especially if they are short. This is because it becomes unlikely that there will be any reversible way of moving, adding, or deleting a key position. Thus, it becomes more important to specify the number of key positions in advance with the *w* parameter.

glam2scan is fast: it can scan a typical motif against the whole Swiss-Prot database in seconds. Its memory requirement scales linearly with the length of the longest individual sequence. Huge sequences such as whole mammalian chromosomes would require massive amounts of memory.

## Discussion

This study demonstrates that a powerful motif discovery method, Gibbs sampling, can be adapted to discover motifs with arbitrary insertions and deletions. Thus, we hope that researchers will not limit themselves to searching for gapless motifs in future. A remarkable point is that glam2 is not substantially slower than the original gapless Gibbs sampler to which it is highly analogous: the big-*O* complexity of the central step, realigning one sequence to the motif, does not change when arbitrary gaps are allowed.

glam2 is most obviously useful for discovering and refining short protein motifs associated with functional sites, such as glycosylation sites, interaction sites, and cleavage sites. These motifs, together with contextual filters such as those used by ELM, should help us to elucidate many of the protein activities that contribute to biological systems. The application of glam2 to protein kinase substrates was somewhat hampered by (i) low availability of non-redundant substrate sequences for each class of kinase and (ii) limited information in current databases and the literature concerning motifs involved with kinase-substrate interaction. However, glam2 was clearly capable of identifying interesting short peptide motifs in sets of sequences related only by their role as substrates of a protein kinase family. This initial study suggests that in combination with other resources, glam2 is a useful tool for analysis of motifs involved in protein-protein interaction.

An exciting but more speculative application of glam2 is discovery of complex gapped motifs in DNA and RNA. Currently known DNA motifs tend to be gapless or bipartite, but it is plausible that multi-factor complexes bind to more complex motifs. Known transcription factor binding motifs are far too non-specific for accurate predictions [56], and complex composite motifs might just supply the needed specificity. RNA molecules frequently contain functional sites with motifs that mediate, for example, subcellular localization and degradation. Myriad functions are emerging for non-coding RNA [57]. While secondary and tertiary structure may be important for many RNA functions, it is likely that sequence motifs will often be present too, just as for the protein motifs in ELM and PROSITE.

While glam2 performs respectably on the BAliBASE multiple alignment benchmark, it is not the best tool for this kind of alignment, except perhaps for motif-like cases with N- and C-terminal extensions. glam2 is not really designed for extensive alignments such as these. Specifically, the following issues probably prevent it from performing better in this assessment:

- glam2's simple motif model is not ideal for protein structural domains, because it does not favour multiple deletions in a row, and perhaps also because it does not distinguish insertion-opening and insertion-extension probabilities. These two properties could be added to our model, making it identical to a profile HMM [58]. We believe that the mathematical development of glam2's scoring scheme and optimisation algorithm (Materials and Methods, Text S1) could be adapted to this more complex model without fundamental difficulties. Better still, perhaps, would be a reticulate (branching) model accommodating partial order alignments (i.e. different subsets of sequences can be aligned to each other in different parts of the alignment) [59].
- glam2's scoring scheme for a column of aligned residues assumes the sequences are equally and distantly related to one another, which is violated by construction in BAliBASE categories (2) and (3). One crude way to address this issue would be a weighting scheme that down-weights highly similar sequences.
- Since glam2 can only adjust the number of key positions by one at a time, it can have difficulty optimising the alignment width, especially if it needs to extend over large insertions. We have no idea how to solve this problem (other than increasing *n*), but we are surprised how well glam2 does on BAliBASE category (5) with large insertions.
- glam2's scoring scheme is based on a Dirichlet mixture, whereas other alignment tools typically use a residue similarity matrix such as BLOSUM-62. Dirichlet mixture priors are more general and potentially more powerful than similarity matrices, but much harder to derive. Thus, we suspect there is more room for improvement in Dirichlet mixtures than in similarity matrices.

In all, we are pleasantly surprised that glam2 is as competitive on this assessment as it is.

It is sometimes desirable to search for multiple motifs, not just the strongest one. This can be accomplished by first obtaining the optimal glam2 motif, then masking the aligned instances of this motif using the companion glam2mask utility, and then re-running glam2.

Since glam2 always reports a motif, even for random sequences, it is often desirable to know whether a motif is statistically significant. Unfortunately this is not easy, but two approaches used with the original Gibbs sampler can be used here too [29]. The first is to run glam2 on multiple shuffled versions of the original sequences, and observe how rarely the motif score for shuffled sequences exceeds the motif score for the original sequences. The second is to concatenate each original sequence with a shuffled version of itself, run glam2 on these hybrid sequences, and check whether the aligned segments occur in the original sequences more often than, or with higher marginal scores than, in the shuffled sequences. The statistical significance can be quantified using a Wilcoxon signed rank test [29]. The second approach is faster, but lacks statistical power when there are few sequences. The tests described here assume that “statistically significant” means “with higher score than likely for randomly shuffled sequences”, which may or may not be appropriate.

We have presented an algorithm to detect similarities across multiple sequences, which bridges the gap between traditional motif discovery methods and multiple alignment techniques. It has a simple and general framework, which seems best suited to subtle, linear motifs with multiple insertions and deletions.

## Supporting Information

Text S1 GLAM2 Methods
(0.14 MB PDF)

Dataset S1 Sets of Sequences Containing PROSITE Motifs
(0.10 MB ZIP)

Dataset S2 Sets of ELM Sites with Flanking Residues
(0.02 MB ZIP)

Dataset S3 GLAM2 Motifs Made from ELM Sites
(0.05 MB ZIP)

Dataset S4 Groups of Protein Kinase Substrate Sequences
(0.11 MB ZIP)
