# Supplementary material for: Discovering Sequence Motifs with Arbitrary Insertions and Deletions
Source: PLoS Comput Biol. 2008 May 9;4(5):e1000071. doi: 10.1371/journal.pcbi.1000071 (PMC2323616; doi:10.1371/journal.pcbi.1000071)
Supplement: Text S1 — GLAM2 Methods (0.14 MB PDF) [file pcbi.1000071.s001.pdf]

GLAM2 Methods

Martin C Frith

August 24, 2007

Overview

The GLAM2 software aims to find the strongest motif that is present in a set of protein or nucleotide sequences. In fact, it can analyze sequences over any user-specified alphabet. The method is described here in three stages: firstly, what exactly is meant by a "motif", secondly, what is meant by "strongest", and finally, the algorithm used to search the space of motifs for the strongest one. Most aspects of the method have close analogs in the well known Gibbs Sampling technique for motif discovery [8, 9, 5]: GLAM2 is essentially a generalization of Gibbs Sampling to allow insertions and deletions in a fully general fashion.

Having found a motif, we often wish to scan it against a database of sequences, to find other instances of the motif. GLAM2 includes such a scanning method, which is also described here.

Motif Definition

A motif in GLAM2 has a certain number, W, of "key positions". The idea is that the key positions hold residues that are important for the motif's function. An instance of the motif is a string of residues (amino acids or nucleotides), where each residue either occupies one of the key positions, or is inserted between key positions. More than one residue may be inserted between key positions, and a key position may lack a corresponding residue, meaning that it is deleted in this motif instance.

What GLAM2 searches for is an alignment of substrings of the input sequences to a series of key positions. The number of key positions is optimized by the algorithm. Currently, each input sequence may contribute at most one substring to the alignment, although this restriction could be relaxed in principle. GLAM2 requires the alignment to contain at least some minimum number of substrings, by default 2. This lower limit is a useful generalization of the OOPS (one occurrence per sequence) and ZOOPS (zero or one occurrence per sequence) modes of previous motif discovery algorithms [2]. For nucleotide sequences, there is an option to consider both strands of the input sequences (direct and reverse-complement).

Scoring Scheme

Overview

In order to define the "strongest" motif (i.e. alignment of substrings to key positions), GLAM2 uses a formula to assign a numeric score to any given alignment. The task, then, is to find the alignment with maximum score. While the precise formula is given below, some desirable characteristics for any sensible formula are noted here. Firstly, the formula should favour alignments where key positions are occupied by identical or chemically similar residues. Secondly, deletions and insertions should be penalized in general. Thirdly, deletions and insertions should be penalized less strongly when they are concentrated in a few positions in the alignment, suggesting that those positions in the motif are more prone to deletion or insertion, than when they are scattered across many positions. The formula used by GLAM2 and described below has each of these characteristics.

Motif Model

The formula is motivated by a simple statistical model of a motif, with position-specific residue probabilities, position-specific deletion probabilities, and position-specific insertion probabilities. Thus, each key position has characteristic probabilities, θ_i, of containing the ith residue type. ($1 \leq i \leq A$, where A is the size of the alphabet, 20 for proteins and 4 for nucleotides.) Also, each key position has probability ϕ of being deleted. Finally, the probability that x residues are inserted between two key positions is $\psi^x(1-\psi)$. The use of a geometric distribution for insertion length is arbitrary, but it is the simplest choice, and since ψ may vary with position, we doubt that more complex schemes would lead to much benefit. This model can be regarded as a hidden Markov model, where θ_i are emission probabilities and ϕ and ψ are transition probabilities.

Hence, in a set of s independent motif instances, the probability that a key position is deleted in a particular subset of d instances and present in the other $m = s - d$ instances is:

$$\phi^d(1-\phi)^m \tag{1}$$

The probability that r residues in total (in all instances) are inserted between two key positions is:

$$\psi^r(1-\psi)^s \tag{2}$$

The probability of observing particular residues in one key position, where c_i is the count of the ith residue type (so $m = \sum_{i=1}^{A} c_i$), is:

$$\prod_{i=1}^{A} \theta_i^{c_i} \tag{3}$$

Motif Priors

The values of θ_i, ϕ, and ψ are, of course, unknown, but prior probability distributions are assigned to these parameters. Then, total probabilities can be obtained by integrating over the parameter values. The total probability of observing d deletions is:

$$\int_0^1 \phi^d (1-\phi)^m \cdot prior(\phi) \mathrm{d}\phi \tag{4}$$

The total probability of observing r insertions is:

$$\int_0^1 \psi^r (1-\psi)^s \cdot prior(\psi) \mathrm{d}\psi \tag{5}$$

The total probability of observing $\vec{c}$ residue counts is:

$$\int \prod_{i=1}^{A} \theta_i^{c_i} \cdot prior(\vec{\theta}) \mathrm{d}\vec{\theta} \tag{6}$$

The prior distributions of ϕ and ψ are assumed to be Beta distributions (the simplest kind of Dirichlet distribution):

$$prior(\phi) = \phi^{D-1}(1-\phi)^{E-1}/Z \tag{7}$$

$$prior(\psi) = \psi^{I-1}(1-\psi)^{J-1}/Z' \tag{8}$$

The values of D, E, I, and J are chosen so as to give GLAM2 the desired level of aversion to deletions and insertions. For reasons that will become clearer in the description of the search algorithm, these parameters are referred to as pseudocounts: D is the deletion pseudocount, E is the no-deletion (or match) pseudocount, I is the insertion pseudocount, and J is the no-insertion pseudocount. Z and Z' are normalization constants. The use of Beta distributions here is ultimately arbitrary, but they are a natural choice since they are conjugate priors of the binomial distributions for deletion and insertion probabilities, and they allow the integrals to be solved analytically. Also, the shapes of these distributions allow considerable flexibility by varying the pseudocount values.

The prior distribution of $\vec{\theta}$ should incorporate prior knowledge of the functional similarities between residues, at least in the case of proteins. To accomplish this, a Dirichlet mixture distribution is used (a weighted sum of Dirichlet distributions):

$$prior(\vec{\theta}) = \sum_{j=1}^{C} \frac{w_j}{Z_j} \prod_{i=1}^{A} \theta_i^{\alpha_{ji}-1} \tag{9}$$

C is the number of components in the mixture, w_j is the weight of the jth component, Z_j is the normalization constant of the jth component, and α_{ji} is the jth component's pseudocount for the ith residue type. So, this distribution

has a potentially huge number of parameters, which should be exquisitely chosen so as to favour biologically realistic values of $\vec{\theta}$. Fortunately, Dirichlet mixture priors for proteins have been extensively investigated by a team at UC Santa Cruz, and we simply use their parameters [11]. For the case of nucleotides, we use a one-component mixture with all pseudocounts = 0.4 [5]. These are the defaults: alternative Dirichlet mixture parameters may be supplied as input to GLAM2.

As far as we can tell, previous implementations of Gibbs Sampling have only used simple Dirichlet priors rather than Dirichlet mixtures, hence have lacked prior information about functional similarities between amino acids. This ought to give GLAM2 a significant advantage in finding protein motifs, even leaving aside the treatment of insertions and deletions.

Using these prior distributions, the total probability integrals can be solved analytically. The solutions contain Gamma functions, denoted by $\Gamma(\cdot)$. The total probability of observing d deletions becomes:

$$\frac{\Gamma(D+E)\Gamma(d+D)\Gamma(m+E)}{\Gamma(s+D+E)\Gamma(D)\Gamma(E)} \tag{10}$$

The total probability of observing r insertions becomes:

$$\frac{\Gamma(I+J)\Gamma(r+I)\Gamma(s+J)}{\Gamma(r+s+I+J)\Gamma(I)\Gamma(J)} \tag{11}$$

The total probability of observing $\vec{c}$ residue counts becomes:

$$\sum_{j=1}^{C} \frac{w_j \cdot \Gamma(A_j)}{\Gamma(m+A_j)} \prod_{i=1}^{A} \frac{\Gamma(c_i+\alpha_{ji})}{\Gamma(\alpha_{ji})} \tag{12}$$

where $A_j = \sum_{i=1}^{A} \alpha_{ji}$.

Background Model

To score alignments, the motif model is compared to a background model of independent sequences. The background model is that the residues occur randomly and independently, with probabilities p_i. For the nucleotide alphabet, the default is $p_i = 1/4$. For the protein alphabet, the amino acid abundances of Robinson and Robinson, which are frequently cited in publications on NCBI BLAST, are used [10, 1]. These probabilities can be adjusted by the user. Thus, the probability of observing $\vec{c}$ residue counts in one key position given the background model is:

$$\prod_{i=1}^{A} p_i^{c_i} \tag{13}$$

In the motif model, residues inserted between key positions are assumed to occur with these same background frequencies.

The Formula

The score S of an alignment X is: the likelihood of the alignment given the motif model, divided by the likelihood of the substrings in the alignment given the background model.

$$
\begin{aligned}
S(X) = \prod_{k=1}^{W} \sum_{j=1}^{C} \frac{w_j \cdot \Gamma(A_j)}{\Gamma(m_k + A_j)} \prod_{i=1}^{A} \frac{\Gamma(c_{ki} + \alpha_{ji})}{\Gamma(\alpha_{ji}) \cdot p_i^{c_{ki}}} \times \\
\prod_{k=1}^{W} \frac{\Gamma(D+E)\Gamma(d_k+D)\Gamma(m_k+E)}{\Gamma(s+D+E)\Gamma(D)\Gamma(E)} \times \\
\prod_{k=1}^{W-1} \frac{\Gamma(I+J)\Gamma(r_k+I)\Gamma(s+J)}{\Gamma(r_k+s+I+J)\Gamma(I)\Gamma(J)}
\end{aligned} \tag{14}
$$

where d_k is the number of deletions of the kth key position, m_k is the number of residues in the kth key position, c_{ki} is the count of the ith residue type in the kth key position, and r_k is the number of insertions between key positions k and $k+1$. Since scores calculated by this formula can easily be of unwieldy orders of magnitude, GLAM2 actually reports $\log_2 S(X)$.

Fitting the Insertion and Deletion Priors

It is desirable to choose the insertion and deletion pseudocounts, D, E, I, and J, so as to maximise the model's fit to actual alignments. Suppose we are given a training set of alignments, with a total of $\mathbf{W}_D$ key positions, and $\mathbf{W}_I$ potential insertion sites between key positions. We seek the values of D, E, I, and J that maximise the total probabilities:

$$
\arg\max_{D,E} \prod_{k=1}^{\mathbf{W}_D} \frac{\Gamma(D+E)\Gamma(d_k+D)\Gamma(m_k+E)}{\Gamma(s_k+D+E)\Gamma(D)\Gamma(E)} \tag{15}
$$

$$
\arg\max_{I,J} \prod_{k=1}^{\mathbf{W}_I} \frac{\Gamma(I+J)\Gamma(r_k+I)\Gamma(s_k+J)}{\Gamma(r_k+s_k+I+J)\Gamma(I)\Gamma(J)} \tag{16}
$$

This can be accomplished, crudely but effectively, by plotting the function over a two-dimensional grid of values for D and E (or I and J). In every case that we examined, the plot indicates a simple hill-shaped function with a unique maximum.

Search Algorithm

Overview

The preceding sections have defined what is meant by a motif: an alignment of substrings of the input sequences to a series of key positions, and have suggested a formula to assign a score to any such alignment. The aim is to find

the alignment(s) with maximum score. Unfortunately, the number of possible alignments will be astronomically huge in most cases, and we do not know a practical algorithm to guarantee finding the highest-scoring one. Therefore it is necessary to use a heuristic algorithm.

GLAM2 uses simulated annealing, a very general technique for finding the highest-scoring solution in a large "search space" of possible solutions. Simulated annealing begins at some (presumably non-optimal) location in the search space, and repeatedly performs stochastic moves to "nearby" locations. Moves that increase the score are favoured, but moves that decrease the score are also permitted, which allows the algorithm to escape from local maxima. A temperature parameter, T, controls the preference for higher scores: at low temperatures they are strongly preferred, but at high temperatures they are only weakly preferred. More precisely, the stochastic moves in simulated annealing should satisfy the so-called detailed balance condition:

$$S(X)^{1/T} P(X \to Y) = S(Y)^{1/T} P(Y \to X) \tag{17}$$

where X and Y indicate locations in the search space, $S(X)$ is the score of location X, and $P(X \to Y)$ is the probability, when in location X, of moving to location Y. Note that detailed balance implies reversibility: if it is possible to move from X to Y, then it must be possible to move from Y to X. The temperature starts out high and is gradually reduced, giving the process a chance to evade local maxima and settle into the global maximum.

The stochastic moves can be generated in many different ways that satisfy detailed balance: generating them in a way that explores the search space effectively is key to a successful algorithm. For example, some previous multiple alignment algorithms have employed simulated annealing with simple moves that adjust the position of a gap in one of the aligned sequences [6, 7]. Thus, each move explores just one location in the vast search space, and many such moves may separate a local optimum from the global optimum. In contrast, GLAM2 uses an extremely clever technique, the *stochastic traceback* [4, 3], which allows a single move to explore efficiently an astronomically large number of alignments (albeit an astronomically small fraction of the total search space).

Moves using the stochastic traceback alone are prone to getting stuck in a certain type of local optimum; so GLAM2 intersperses these with a second type of move. The stochastic traceback moves are referred to as site sampling, and the second type as column sampling, because they are analogous to procedures with the same names in the original Gibbs sampler [9]. At each iteration, one type of move is chosen randomly with 50:50 probabilities. A modification of column sampling allows the number of key positions in the motif to be varied. Together, these moves can explore the space of all possible motifs.

To assess the robustness of the result, GLAM2 performs several (by default 10) annealing runs from different random starting points. Each run continues until some number of iterations n has passed without finding a higher-scoring alignment. During each run, T is reduced by a fixed percentage at each iteration, by default starting at 1.2 and decreasing 1.44-fold per n iterations. The highest-scoring alignment encountered in each run is reported at the end.

Site Sampling

Overview

In site sampling, one of the input sequences is chosen at random and re-aligned to the motif. All possible alignments of substrings of this sequence to the motif are considered, including the option of excluding this sequence from the alignment altogether. However, if excluding the sequence would bring the number of substrings in the alignment below the minimum permitted number (see above), then exclusion is not considered. Both strands of the sequence are considered, if the user selected this option. One alignment is chosen at random, with probability proportional to the resulting alignment score, as defined above, raised to the power of $1/T$.

Scoring

The calculations can be simplified somewhat by dividing by a constant factor: the score of the alignment of all the other sequences, excluding the sequence that is being re-aligned. (This also scales the values to moderate orders of magnitude that are likely to be representable by the computer.) In the following, primed letters refer to this alignment of all the other sequences: s' is the number of substrings in the alignment, m'_k is the number of residues aligned to key position k, d'_k is the number of deletions of key position k, r'_k is the number of insertions between k and $k+1$, and c'_{ki} is the count of residue type i in position k.

Using the fact that $\Gamma(x) = (x-1)\Gamma(x-1)$, the score for deleting key position k (cf. Equation 10) becomes:

$$\delta(k) = \frac{d'_k + D}{s' + D + E} \tag{18}$$

The score for inserting x residues between k and $k+1$ (cf. Equation 11) becomes:

$$\iota(k,x) = \frac{s' + J}{r'_k + s' + I + J} \cdot \prod_{i=0}^{x-1} \frac{r'_k + I + i}{r'_k + s' + I + J + i + 1} \tag{19}$$

The score for aligning a residue of type X with key position k (cf. Equation 12) becomes:

$$\mu(k,X) = \frac{m'_k + E}{s' + D + E} \cdot \sum_{j=1}^{C} v_{kj} \frac{c'_{kX} + \alpha_{jX}}{m'_k + A_j} \Big/ p_X \tag{20}$$

where:

$$v_{kj} = \frac{\frac{w_j \cdot \Gamma(A_j)}{\Gamma(m'_k + A_j)} \prod_{i=1}^{A} \frac{\Gamma(c'_{ki} + \alpha_{ji})}{\Gamma(\alpha_{ji})}}{\sum_{j=1}^{C} \frac{w_j \cdot \Gamma(A_j)}{\Gamma(m'_k + A_j)} \prod_{i=1}^{A} \frac{\Gamma(c'_{ki} + \alpha_{ji})}{\Gamma(\alpha_{ji})}} \tag{21}$$

(Hence D, E, I, and J are called pseudocounts, because they get added to the observed counts: d'_k, m'_k, r'_k, and s'.)

Dynamic Programming

To set up the stochastic traceback, GLAM2 calculates values $M(i,j)$: the sum of the scores of all alignments ending at the ith key position in the motif and the jth residue in the sequence. The calculation is made slightly simpler by constructing intermediate values $N(i,j)$. The quantities μ, δ, and ι are raised to the power of $1/T$ prior to using them in this procedure. In the following, L is the length of the sequence. Boundary cases:

$$N(0,j) = 1 \qquad (0 \leq j \leq L) \tag{22}$$

$$M(i,0) = \delta(i) \cdot N(i-1,0) \qquad (1 \leq i \leq W) \tag{23}$$

Main cases:

$$M(i,j) = \mu(i,X_j) \cdot N(i-1,j-1) + \delta(i) \cdot N(i-1,j) \qquad \begin{pmatrix} 1 \leq j \leq L \\ 1 \leq i \leq W \end{pmatrix} \tag{24}$$

$$N(i,j) = \sum_{x=0}^{j} \iota(i,x) \cdot M(i,j-x) \qquad \begin{pmatrix} 0 \leq j \leq L \\ 1 \leq i < W \end{pmatrix} \tag{25}$$

This procedure is similar to the standard algorithms for pair-wise sequence alignment, but much slower: the summation in Equation (25) causes the whole calculation to require $O(WL^2)$ operations rather than $O(WL)$ operations. Ways to speed up the calculation are described below.

Stochastic Traceback

The values $M(i,j)$ are used to pick a random alignment, with probabilities proportional to their scores to the power of $1/T$. Note that $\sum_{j=0}^{L} M(W,j)$ is the sum of the scores of all alignments of the sequence to the motif. If both strands are being considered, a second matrix $M_R(i,j)$ is constructed using the reverse complement of the sequence. GLAM2 randomly decides to align the sequence on the forward strand, the reverse stand, or not at all, with probabilities in the ratio $\sum_{j=0}^{L} M(W,j) : \sum_{j=0}^{L} M_R(W,j) : 1$. Supposing the forward strand is chosen, the endpoint of the alignment in the sequence, $0 \leq j \leq L$, is then picked randomly, with probabilities proportional to $M(W,j)$.

Having chosen the endpoint j, the alignment is determined by iterating the following steps, with i initially equal to W. Firstly, GLAM2 randomly chooses to either align residue j to key position i, or delete key position i, with probabilities defined in Equations (26) and (27). Then i is decremented, and unless deletion was chosen, j is decremented. Secondly, GLAM2 randomly picks a number of residues, $0 \leq x \leq j$, to insert between key positions i and $i+1$, with probabilities given in Equation (28). x is then subtracted from j. These steps are repeated until i or j reaches zero.

$$\text{prob(match)} = \mu(i,X_j) \cdot N(i-1,j-1) \; / \; M(i,j) \tag{26}$$

$$\text{prob(delete)} = \delta(i) \cdot N(i-1,j) \; / \; M(i,j) \tag{27}$$

$$\text{prob(insert } x \text{ residues)} = \iota(i,x) \cdot M(i,j-x) \; / \; N(i,j) \tag{28}$$

The traceback can be performed in $O(W + L)$ operations.

Faster Algorithms

GLAM2 provides two options to speed up the dynamic programming procedure. The first option, noting that Equation (25) is a convolution, is to calculate it in $O(L \log L)$ operations using fast Fourier transforms. Thus, the whole dynamic programming procedure requires $O(WL \log L)$ operations. A potential disadvantage is loss of accuracy for smaller values of $N(i, j)$. The values of $M(i, j)$ and $N(i, j)$ (for fixed i) may differ by many orders of magnitude; since the Fourier transform mixes these values, the smaller ones become corrupted owing to limited precision of the computer's representation of real numbers. Unfortunately, in some cases these smaller values of $N(i, j)$ have large effects at later stages of the dynamic programming.

The second option is to replace the residue insertion score, $\iota(k, x)$, with a more convenient approximation:

$$\iota(k, x) = \iota_1(k) \cdot \iota_2(k)^x \tag{29}$$

where:

$$\iota_1(k) = \frac{s' + J}{r'_k + s' + I + J} \tag{30}$$

$$\iota_2(k) = \frac{r'_k + I}{r'_k + s' + I + J} \tag{31}$$

Here, ι_2 and ι_1 are the posterior mean estimators for ψ and $1 - \psi$. With this change, $N(i, j)$ can be built up as follows:

$$N(i, 0) = \iota_1(i) \cdot M(i, 0) \tag{32}$$

$$N(i, j) = \iota_1(i) \cdot M(i, j) + \iota_2(i) \cdot N(i, j - 1) \tag{33}$$

Thus, the whole dynamic programming procedure requires $O(WL)$ operations. This is the default algorithm for GLAM2. It is remarkable that this algorithm samples from all gapped alignments using the same number of operations, $O(WL)$, as the site sampling step of the original Gibbs sampler [8], which only considers ungapped alignments. The change to $\iota(k, x)$ introduces a slight deviation from detailed balance, penalizing long insertions, but seems to produce sensible alignments in practice.

Numerical Considerations

The values of $M(i, j)$ and $N(i, j)$ can become too large or too small to be represented by the computer using floating point. A rescaling procedure is employed to mitigate this problem. After calculating $N(i - 1, j)$ for all j, and

before calculating $M(i,j)$, GLAM2 rescales $\mu(i,X)$ and $\delta(i)$ as follows:

$$\mu(i,X) \leftarrow \frac{\mu(i,X)}{\max_{j=0}^{L} N(i-1,j)} \tag{34}$$

$$\delta(i) \leftarrow \frac{\delta(i)}{\max_{j=0}^{L} N(i-1,j)} \tag{35}$$

The product of the rescale values, R, is recorded (in log space to avoid overflow):

$$R = \prod_{i=1}^{W} \max_{j=0}^{L} N(i-1,j) \tag{36}$$

In the stochastic traceback, the sum of alignment scores $\sum_{j=0}^{L} M(W,j)$ needs to multiplied by R, and similarly for M_R, before picking the strand. Note that this method is simpler than rescaling procedures that have been described elsewhere [3]: there is no need to record an array of rescaling parameters, and the traceback, after picking the strand, works properly without change.

This rescaling keeps the maximum values of $M(i,j)$ and $N(i,j)$ to manageable orders of magnitude, but the minimum values may still underflow. These small values "usually" correspond to extremely improbable alignments, but in slightly pathological cases they can be the seeds of highly probable alignments. GLAM2 attempts to warn when this occurs, by checking whether the traceback passes through values of $N(i,j)$ below the limit of accurately represented numbers (DBL_MIN). As noted above, the fast Fourier transform algorithm entails a more severe loss of accuracy for small values: in this case, the warning is triggered at values below DBL_EPSILON. In addition, illegitimate negative values of $N(i,j)$ emerging from the fast Fourier transform are immediately reset to zero.

Finally, problems can occur with low values of T. For instance, an entire row of $N(i,j)$ values (all j for some i) can underflow to zero. To avoid this, a lower bound is imposed on T, by default 0.1.

Column Sampling

Motivation

Site sampling alone finds high-scoring alignments, but often with non-optimal distribution of the key positions. For example, there might be a key position that is deleted in all substrings, while further along the alignment there is a column of inserted residues that are all identical. In this case, the score would increase if all the letters between these two columns were shifted across by one position, so that the identical residues become aligned to a key position, and the deletions disappear. Unfortunately, site sampling moves can only shift the letters of one substring at a time, which is likely to decrease the score. So low-scoring intermediate alignments must be traversed in order to reach the improved alignment. Column sampling moves solve this difficulty by providing a more direct route to the improved alignment.

Overview

Column sampling has two steps. In the first step, one key position is chosen at random, and removed from the alignment. In the second step, a new key position is added to the alignment. These steps can be viewed as moving a key position from one location to another in the alignment.

Removal of a key position has the following effect. If an internal key position (neither the leftmost nor the rightmost) is removed, the residues that were aligned to it become insertions between the preceding and following key positions. If the leftmost or rightmost key position is chosen, the residues that were aligned to it cease to be part of the alignment.

In general, the number of ways of adding a key position to an alignment is vast, and the addition step needs to be restricted to a manageable subset of these. Furthermore, this subset should be chosen in a way that ensures reversibility and detailed balance of column sampling moves. To achieve this, some properties of the key position that was removed need to be preserved.

Details

Before removing a key position, the following information is recorded about it: whether it is deleted or matched in each substring, and the number of insertions in each substring between this key position and the one to either the left or the right. The direction, left or right, is chosen randomly with 50:50 probability before choosing the key position. When the direction is "left", the leftmost key position is never chosen, and when the direction is "right", the rightmost key position is never chosen. This procedure requires that there are at least two key positions.

After removing the key position, GLAM2 considers all ways of adding a key position with these properties to the alignment. The numbers of insertions between it and the neighbouring key position are preserved relatively rather than absolutely: in other words, they may be shifted by a constant offset. The alignment score for each way of adding such a key position is calculated, and one of them is chosen randomly with probabilities proportional to their scores raised to the power of $1/T$.

Changing the number of key positions

Overview

None of the moves described so far changes the number of key positions in the motif, yet we wish to optimise this also. This is accomplished by two modifications to the column sampling procedure. Firstly, in the removal step, a key position is chosen and its properties are recorded as usual, but it is not always removed. This allows the number of key positions to grow. The decision to remove or not is made stochastically, with probability q of removal. The correct value for q is discussed below. Secondly, in the addition step, a key position is not always added. This allows the number of key positions to decrease.

Modified Addition Step

Suppose that a key position was removed, and that there are no other key positions with the same properties. If we do not add a key position, there will be no way to reverse the move, in violation of detailed balance. Thus, a key position must always be added, unless there is another key position in the alignment with the same properties. One way to think about this is that the key position to be added can be "absorbed" by a key position with the same properties. Specifically, GLAM2 counts the number, V, of key positions with the same properties as the one being added, and adds absorption by each one of them to the list of alignments that it considers. Finally, as usual, one alignment is chosen stochastically with probability proportional to the alignment score to the power of $1/T$.

Probability of Removal

The probability q of removal should be chosen carefully to maintain detailed balance. Consider the probabilities of moving between two alignments X and Y, where Y has one extra key position compared to X. In the following, V_X is the number of key positions in X with the same properties as the extra key position in Y.

$$P(X \to Y) = \frac{1}{2} \cdot \frac{1}{2} \cdot (1 - q(X)) \cdot \frac{V_X}{W_X - 1} \cdot \frac{S'(Y)}{V_X \cdot S'(X) + \sum_Z S'(Z)} \tag{37}$$

where W_X is the number of key positions in X (one of which, either the leftmost or rightmost, is never selected), $S'(X)$ is the score of X to the power of $1/T$, and Z sums over all the ways of adding a new key position. The first $1/2$ is the probability of column sampling rather than site sampling; the second $1/2$ is the probability of choosing the direction (left or right); $1 - q(X)$ is the probability of not removing the key position; $V_X/(W_X - 1)$ is the probability of choosing a key position with the right properties to get to Y; the final fraction is the probability of adding a key position in the right location to get to Y. Similarly:

$$P(Y \to X) = \frac{1}{2} \cdot \frac{1}{2} \cdot q(Y) \cdot \frac{1}{W_Y - 1} \cdot \frac{V_X \cdot S'(X)}{V_X \cdot S'(X) + \sum_Z S'(Z)} \tag{38}$$

Thus, to achieve detailed balance, the following must hold:

$$\frac{1 - q(X)}{W_X - 1} = \frac{q(Y)}{W_Y - 1} \tag{39}$$

where $W_X = W_Y - 1$. This can be satisfied if q is a function of the number of key positions, W. To simplify things a bit, we express q as a function of the number of *selectable* key positions, $W' = W - 1$, recalling that one of the two

endmost key positions is never selected. Then:

$$\frac{q(W'+1)}{W'+1} = \frac{1}{W'} - \frac{q(W')}{W'} \tag{40}$$

$$\Rightarrow \frac{q(W')}{W'} = (-1)^{W'-1}\left[q(1) - \sum_{i=1}^{W'-1} \frac{(-1)^{i-1}}{i}\right] \tag{41}$$

The summation in Equation (41) generates the Taylor expansion of $ln(2)$ about 1, so the unique solution that prevents $q(W')$ from diverging is, rather surprisingly, $q(1) = ln(2)$.

Initialization

At the start of each annealing run, an initial alignment is constructed in the following manner. The number of key positions is set to an initial value chosen by the user (default: 20). Starting with an empty alignment, the input sequences are taken one-by-one, in a random order, and added to the alignment using a site sampling move with $T = 1$.

Motif Scanning

Overview

In motif scanning, a sequence database is searched to find new instances of a previously determined motif. An instance is a sequence segment that has a high-scoring alignment to the key positions of the motif. In this section, we first describe the scoring scheme for such alignments. Secondly, we describe an algorithm to scan a motif against one sequence, and find a guaranteed maximal-scoring alignment. To scan a database, this algorithm can simply be repeated for each sequence. However, some sequences may contain multiple motif instances: we do not want to be limited to one hit per sequence. Thus, we also describe a method for finding suboptimal matches. The techniques used in this section are all standard [3].

Scoring Scheme

Motif Model

The scoring scheme derives from the statistical motif model described earlier. To recap, the model has position-specific residue probabilities: θ_i, deletion probabilities: ϕ, and insertion probabilities: $\psi^x(1-\psi)$. For scanning, these probabilities are estimated from the predetermined motif, and from prior expectations. Specifically, the posterior mean estimators [3] are used.

We are given a motif with W key positions and s aligned sequences. The kth key position has m_k residues and is deleted d_k times, so that $m_k + d_k = s$.

There are r_k insertions between key positions k and $k+1$. c_{kX} is the count of residue type X in key position k, so that $m_k = \sum_{X=1}^{A} c_{kX}$. Using the same Beta and Dirichlet mixture priors as above, the posterior mean estimators are:

$$\hat{\phi}(k) = \frac{d_k + D}{s + D + E} \tag{42}$$

$$\hat{\psi}(k) = \frac{r_k + I}{r_k + s + I + J} \tag{43}$$

$$\hat{\theta}(k, X) = \sum_{j=1}^{C} v_{kj} \frac{c_{kX} + \alpha_{jX}}{m_k + A_j} \tag{44}$$

where:

$$v_{kj} = \frac{\frac{w_j \cdot \Gamma(A_j)}{\Gamma(m_k + A_j)} \prod_{i=1}^{A} \frac{\Gamma(c_{ki} + \alpha_{ji})}{\Gamma(\alpha_{ji})}}{\sum_{j=1}^{C} \frac{w_j \cdot \Gamma(A_j)}{\Gamma(m_k + A_j)} \prod_{i=1}^{A} \frac{\Gamma(c_{ki} + \alpha_{ji})}{\Gamma(\alpha_{ji})}} \tag{45}$$

Background Model

We evaluate a potential motif instance by comparing the motif model to a background model. As before, the background model is that the residues occur randomly and independently, with probabilities p_i. The p_i values can be adjusted by the user. By default, For proteins, the abundances of Robinson and Robinson are used [10], and for nucleotides, uniform abundances are used.

Score Parameters

The score of a sequence segment aligned to a motif is a log likelihood ratio: the likelihood of the aligned segment given the motif model, versus the likelihood of the segment given the background model. Thus, the score for deleting key position k is:

$$\delta(k) = \log_2 \left[\hat{\phi}(k) \right] \tag{46}$$

The score for aligning a residue of type X with key position k is:

$$\mu(k, X) = \log_2 \left[(1 - \hat{\phi}(k)) \cdot \hat{\theta}(k, X) \ / \ p_X \right] \tag{47}$$

The score for inserting x residues between k and $k+1$ is $\iota_1(k) + \iota_2(k) \cdot x$, where:

$$\iota_1(k) = \log_2 \left[1 - \hat{\psi}(k) \right] \tag{48}$$

$$\iota_2(k) = \log_2 \left[\hat{\psi}(k) \right] \tag{49}$$

Search Algorithm

Overview

We wish to find a maximal-scoring alignment of the motif's key positions with a segment of the query sequence. This is a standard alignment problem, which can be solved by dynamic programming followed by a traceback.

Dynamic Programming

Dynamic programming calculates values $M(i,j)$: the score of the highest-scoring alignment ending at the ith key position in the motif and the jth residue in the sequence. The calculation is made slightly simpler by constructing intermediate values $N(i,j)$. In the following, L is the length of the sequence. Boundary cases:

$$N(0,j) = 0 \qquad (0 \le j \le L) \tag{50}$$
$$M(i,0) = \delta(i) + N(i-1,0) \qquad (1 \le i \le W) \tag{51}$$
$$N(i,0) = \iota_1(i) + M(i,0) \qquad (1 \le i < W) \tag{52}$$

Main cases:

$$M(i,j) = \max\begin{cases} \mu(i,X_j) + N(i-1,j-1) \\ \delta(i) + N(i-1,j) \end{cases} \qquad \begin{pmatrix} 1 \le j \le L \\ 1 \le i \le W \end{pmatrix} \tag{53}$$
$$N(i,j) = \max\begin{cases} \iota_1(i) + M(i,j) \\ \iota_2(i) + N(i,j-1) \end{cases} \qquad \begin{pmatrix} 1 \le j \le L \\ 1 \le i < W \end{pmatrix} \tag{54}$$

Traceback

In this section, the values $M(i,j)$ and $N(i,j)$ are used to find a highest-scoring alignment. First, a highest-scoring alignment endpoint is found:

$$j = \arg\max_{j=0}^{L} M(W,j) \tag{55}$$

In case of ties, the lower value of j is arbitrarily chosen.

Having chosen the endpoint j, the alignment is determined by iterating the following steps, with i initially equal to W. Firstly, if $\delta(i)+N(i-1,j)$ is greater than $\mu(i,X_j)+N(i-1,j-1)$, key position i is deleted, else residue j is aligned to key position i. Then i is decremented, and unless deletion was chosen, j is decremented. Secondly, if $\iota_2(i) + N(i,j-1)$ is greater than $\iota_1(i) + M(i,j)$, a residue is inserted between key positions i and $i+1$, and j is decremented. This is repeated until $\iota_2(i) + N(i,j-1)$ ceases to be greater than $\iota_1(i) + M(i,j)$. These steps are repeated until i or j reaches zero.

Suboptimal Matches

Overview

The previous algorithm finds one highest-scoring alignment of a motif to a sequence, but we wish to be able to find more than one alignment. The usual definitional problem arises: there are a vast number of possible alignments, and the second-highest scoring one is likely to be a minor variant of the highest-scoring one, but we are not interested in such variants. Thus, we adopt the criterion of Waterman and Eggert [12]: subsequent alignments must not pair residues and key positions that have been paired in any previous alignment.

Method

To satisfy this criterion, we maintain a matrix $F(i, j)$ that indicates forbidden pairs. $F(i, j)$ is initialized to all zeros. During the traceback, whenever residue j is aligned to key position i, $F(i, j)$ is set to 1. After the traceback, the M and N matrices are recalculated, with matches forbidden whenever $F(i, j) = 1$. Only small regions of M and N near the newly forbidden cells need to be recalculated [12]. Finally, a new traceback is performed, avoiding old forbidden matches, and marking new forbidden matches. These recalculation and traceback steps can be repeated as often as desired.

Stopping Criteria

Retrieval of suboptimal matches from one sequence stops when either of two conditions are met. Firstly, if an alignment with no matches (only deletions and insertions) is found, it is discarded and the procedure is terminated. Such an alignment does not add any forbidden pairings, so it necessarily terminates the Waterman-Eggert procedure. Secondly, the total number of database hits is limited to some value n chosen by the user. If n hits have already been found, and the new alignment has a score no greater than any of these, it is discarded and the procedure is terminated. The database hits are stored in a heap, which is a kind of partially-sorted array that allows efficient removal of the lowest-scoring hit and insertion of new hits.

References

[1] S F Altschul, T L Madden, A A Schaffer, J Zhang, Z Zhang, W Miller, and D J Lipman. Gapped BLAST and PSI-BLAST: a new generation of protein database search programs. *Nucleic Acids Res*, 25(17):3389–402, 1997.

[2] T. L. Bailey and C. Elkan. The value of prior knowledge in discovering motifs with meme. *Proc Int Conf Intell Syst Mol Biol*, 3:21–29, 1995.

[3] Richard Durbin, Sean R. Eddy, Anders Krogh, and Graeme Mitchison. *Biological sequence analysis: probabilistic models of proteins and nucleic acids.* Cambridge Univ. Press, 2000.

[4] S. R. Eddy. Multiple alignment using hidden markov models. *Proc Int Conf Intell Syst Mol Biol*, 3:114–20, 1995.

[5] Martin C Frith, Ulla Hansen, John L Spouge, and Zhiping Weng. Finding functional sequence elements by multiple local alignment. *Nucleic Acids Res*, 32(1):189–200, 2004.

[6] M Ishikawa, T Toya, M Hoshida, K Nitta, A Ogiwara, and M Kanehisa. Multiple sequence alignment by parallel simulated annealing. *Comput Appl Biosci*, 9(3):267–73, 1993.

[7] J Kim, S Pramanik, and M J Chung. Multiple sequence alignment using simulated annealing. *Comput Appl Biosci*, 10(4):419–26, 1994.

[8] C E Lawrence, S F Altschul, M S Boguski, J S Liu, A F Neuwald, and J C Wootton. Detecting subtle sequence signals: a Gibbs sampling strategy for multiple alignment. *Science*, 262(5131):208–14, 1993.

[9] A F Neuwald, J S Liu, and C E Lawrence. Gibbs motif sampling: detection of bacterial outer membrane protein repeats. *Protein Sci*, 4(8):1618–32, 1995.

[10] A B Robinson and L R Robinson. Distribution of glutamine and asparagine residues and their near neighbors in peptides and proteins. *Proc Natl Acad Sci U S A*, 88(20):8880–4, 1991.

[11] K Sjolander, K Karplus, M Brown, R Hughey, A Krogh, I S Mian, and D Haussler. Dirichlet mixtures: a method for improved detection of weak but significant protein sequence homology. *Comput Appl Biosci*, 12(4):327–45, 1996.

[12] M. S. Waterman and M. Eggert. A new algorithm for best subsequence alignments with application to trna-rrna comparisons. *J Mol Biol*, 197(4):723–728, Oct 1987.
